# Diabetes Burden in Urban and Rural Senegalese Populations: A Cross-Sectional Study in 2012

**DOI:** 10.1155/2015/163641

**Published:** 2015-09-30

**Authors:** S. M. Seck, D. G. Dia, D. Doupa, A. Diop-Dia, I. Thiam, M. Ndong, L. Gueye

**Affiliations:** ^1^Faculty of Health Sciences, Gaston Berger University, Saint-Louis, Senegal; ^2^Nutrition Department, Faculty of Agronomic Sciences, Gaston Berger University, Saint-Louis, Senegal

## Abstract

Diabetes represents a challenging global health issue in the 21st century. Data from sub-Saharan African populations are scarce and are usually restricted to urban settings. The objective of this study was to compare prevalence and risk factors of diabetes in rural and urban areas in Senegal.* Methods.* In a community-based survey between January and May 2012, we included 1027 adults aged ≥18 years living in northern Senegal. Sociodemographic, clinical, and biological data were collected during household visits. Multivariate logistic regression was performed to identify factors associated with diabetes.* Results.* Mean age of participants was 48.0 ± 16.9 years and 65.7% were female. Participants from urban area represented 55.7%. The age-standardized prevalence of diabetes was 7.6% (6.0% in men versus 9.0% in women). Prevalence of diabetes was higher in urban areas (8.1%) compared to rural areas (4.6%). Disease awareness rate was 43%. After multivariate analysis, age (OR = 1.63, *p* = 0.001), familial history of diabetes (OR = 1.42, *p* = 0.001), and abdominal obesity (OR = 1.17, *p* = 0.05) were associated with diabetes.* Conclusion.* Diabetes is frequent in urban and rural areas in Senegal. Awareness rate is very low among populations. Age, family history of diabetes, and abdominal obesity are the main risk factors identified.

## 1. Introduction

Diabetes represents a challenging health issue in the 21st century with a growing incidence estimated to be 381.8 million patients globally in 2013 and high morbidity and mortality rates [1]. Though population data are often scarce, the African continent is expected to bear the most important burden of diabetes during the next coming decades [1–3]. However, a few countries have developed national strategies to contain this coming epidemic [2]. In many cases, lack of data is the major barrier for setting up efficient programs for prevention and management of diabetes in disadvantaged populations. In Senegal, there is not yet available data on diabetes burden at national level but one recent survey in Saint-Louis city (northern region) reported a prevalence of 10.4% with two-thirds of patients uncontrolled [4]. Moreover, marked disparities had been reported between urban and rural regions where lifestyle habits and access to care are different [2–4].

The objective of this study was to compare prevalence and risk factors of diabetes in adult populations living in rural and urban areas in Senegal.

## 2. Population and Methods

### 2.1. Study Design

We performed a community-based cross-sectional survey in Saint-Louis (northern region of Senegal). All individuals aged ≥18 years and living in Saint-Louis for ≥ 3 months were eligible to participate in the study.

### 2.2. Sampling Procedure

A two-stage cluster sampling method was used to select a representative sample of adults living in urban and rural areas of Saint-Louis. We firstly selected 17 localities as clusters (9 urban areas and 8 rural areas). Then, we randomly took a number of households proportional to population size of each locality (data available from the National Agency of Statistics and Demography). From each household a maximum of two participants were randomly recruited among those present at the day of visit.

Considering *α*-error of 0.05 and a power *β* of 80%, the required sample size was 855 individuals and we added a 20% attrition rate to get a sample of 1026 participants. Finally, a total of 1056 persons were sampled to enter the study.

### 2.3. Data Collection

Data were collected on site during house-to-house visits that were conducted between 7 a.m. and 12 p.m. or at the nearest health centre when patients did not live far away from this facility.

A modified version of the WHO STEPwise questionnaire (http://www.who.int/chp/steps/) was pretested and validated before its use to collect data. Researchers assisted by medical students, trained nurse practitioners, and community health workers had to fill the data collection form, to document the sociodemographic status (age, sex, marital status, education, profession, and education), personal and family health history (regarding, particularly, hypertension, diabetes, stroke, and heart and kidney disease), and lifestyle (nutritional habits, physical activity, smoking, and alcohol consumption) of each participant. Anthropometric measurements (weight, height, waist, and hip circumference) were performed using standard methods and calibrated devices. Blood pressure was measured twice at five minutes intervals by a semiautomatic sphygmomanometer and the mean of the two readings was calculated. If the difference between the readings was greater than 10 mm Hg, a third measurement was performed.

Hypertension was defined as a systolic blood pressure of 140 mm Hg or more, diastolic blood pressure of 90 mm Hg, any prescription of antihypertensive medication in the past two weeks, or any self-reported history of hypertension [5]. Obesity was defined using International Diabetes Foundation cut-offs [6].

Serum total cholesterol, LDL cholesterol, HDL cholesterol, and triglycerides were measured with colorimetric method. Fasting blood glucose (FBG) was measured with a glucose oxidase method, and serum total cholesterol, HDL cholesterol, and total triglycerides were measured by an enzymatic calorimetric method. LDL cholesterol was calculated by the Friedewald formula. Diabetes was defined as FBG ≥ 126 mg/dL or by prescription of hypoglycemic agents despite fasting plasma glucose or any self-reported history of diabetes [6].

Physical inactivity was defined as less than 30 minutes of moderate activity per week or less than 20 minutes of vigorous activity three times per week, or the equivalent.

### 2.4. Statistical Analysis

Statistical analyses were performed with STATA 12.0 (Stata Corp, TX, USA). Continuous variables were presented as mean ± standard deviation and categorical variables as percentage. Comparison of proportions and means were done using Pearson's Chi-square test or Student's *t*-test as appropriated. The age-standardized prevalence rates were calculated with the direct method, using the results of Senegalese general population census as the standard (http://www.ansd.sn/). Clinical and biochemical parameters associated with diabetes were assessed, in bivariate analysis, comparing the group with diabetes and the group without diabetes using Student's *t*-test or a Chi-square test. Variables significantly associated with diabetes were then included in a multivariate logistic regression model with age, gender, and urbanization as forced variable. Odds ratio (ORs) with 95% CI and *p* values of the final model are presented.

## 3. Results

A total of 1056 participants were involved in the study with a response rate of 99.1%. But 21 of them were excluded from the analysis because of incomplete collected data and 1026 individuals were finally analyzed. The majority of patients (55.3%) lived in urban area. The main clinical and biochemical characteristics are presented in Table 1.

**Figure 1:**
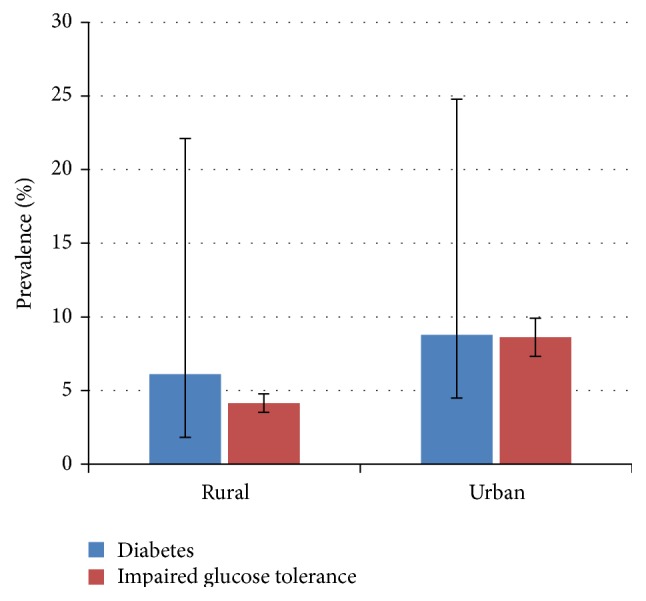
Prevalence of diabetes and impaired glucose tolerance in different settings.

**Figure 2:**
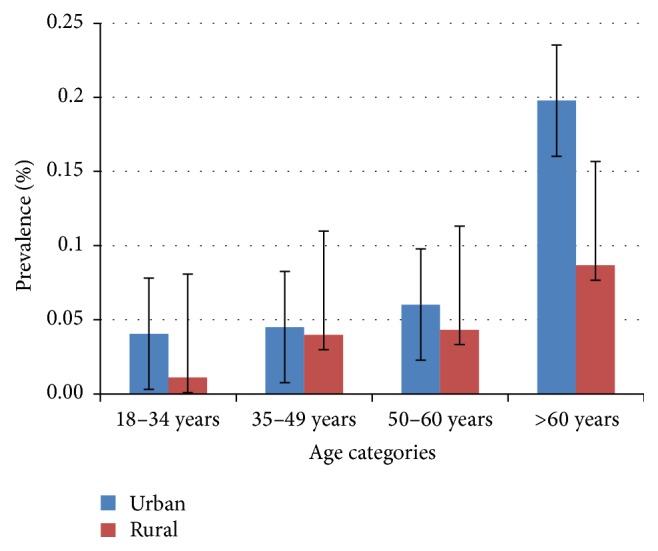
Age-specific prevalence of diabetes in urban and rural areas.

The crude prevalence of diabetes in our sample was 10.8% (95% CI: 6.9%–14.2%). The age-adjusted prevalence of diabetes was 7.6% (95% CI: 5.4%–10.5%) with a difference between men (6.0%) and women (9.0%). The mean age of diabetic patients was 46.8 ± 13.5 years (18–76 years). The crude prevalence of diabetes in urban and rural settings was, respectively, 12.7% and 6.8%. Adjusted prevalence of diabetes was higher in urban areas (8.1%) compared to rural areas (4.6%) (Figure 1). Also, in both settings, there was an increase in diabetes prevalence with age as shown in Figure 2. Comparing cardiovascular risk of people living in urban and in rural settings, we found similarly high prevalence of traditional cardiovascular risk factors such as hypertension, obesity, and physical inactivity contrasting with low proportion of smokers.

Regarding the disease awareness, 43% of patients were diagnosed during the survey, 36% were previously declared diabetic but without any medical follow-up, and 21% were regularly treated by a physician.

The presence of diabetes was associated with common risk factors like age, gender, obesity, and familial history of diabetes (see Table 2). Univariate analysis showed that age ≥35 years (10% of diabetics versus 5.5% in people aged <35 years), female gender (9.0% of diabetics versus 6.0% in males), family history of diabetes (diabetes prevalence of 8.4% versus 5.1%), and obesity (diabetes prevalence of 13.9% versus 8.3%) were significantly associated with a higher chance to get diabetes. Conversely, physical activity and daily consumption of ≥3 fruits/vegetables were associated with lower risk of diabetes in this population (prevalence differences of 19.5% and 4.4%, resp.). After adjustment for age, gender, smoking, familial history, and obesity, the risk of diabetes was similar in individuals living in urban area compared to those living in rural areas (they presented a diabetes risk excess of 27%). Only age > 35 years (OR = 1.63, 95% CI = [1.48–2.06]), existence of family history of diabetes (OR = 1.42, 95% CI = [1.12–3.77]), and abdominal obesity (OR = 1.17, 95% CI = [1.00–1.78]) remained significantly associated with diabetes (see Table 3).

## 4. Discussion

Prevalence of diabetes in sub-Saharan Africa is variable across countries, ethnic groups, and settings considered. Reported data from community-based studies range from 2.8% in rural populations in Angola [7] to 28.2% found in South African mixed ancestry populations living in urban areas [8, 9].

Epidemiological studies in rural populations are scarcer but they generally show a lower prevalence [10–12] compared to surveys in urban settings [13–15].

In this study, the prevalence of diabetes in urban areas is quite twice the one in rural areas. Comparable prevalence ratios (urban/rural) were found in Kenya [12] and in Democratic Republic of Congo [15].

As already reported in the literature, we found an increasing prevalence with age which is a major risk factor for type 2 diabetes [2].

Gender distribution of diabetes is also variable. Some studies found higher prevalence in men [10, 11] and others reported the contrary [9, 16].

In the present study, female gender, absence of school education, and living in urban setting were associated with diabetes at univariate analysis. However, these associations were no longer significant after adjustment for true risk factors that were abdominal obesity, age, and familial history of diabetes.

As underlined in many studies, we found a higher prevalence of diabetes in women compared to men and this difference was more striking among rural populations.

The level of disease awareness is low in our study. However, these rates are better than what was reported in rural Tanzanians (8.3 to 13.2%) [17, 18] or South Africans (15.3%) [19] or in a previous survey in Dakar (capital city) where 90% of newly diagnosed diabetics were not aware of their disease [16].

Recent forecast suggests an alarming increase of diabetes incidence in Africa during the next decade in addition to other noncommunicable and infectious diseases [3]. In the US population between 1980 and 2011, the crude prevalence of diagnosed diabetes increased from 2.5% to 6.9% while age-adjusted prevalence rose in the same proportions indicating that changes in the population age structure do not explain the epidemic transition [20].

The true explanation of this rising burden of diabetes in both urban and rural Africa is probably multifactorial. With life expectancy increase, the most important part might be driven by lifestyle modifications (fast urbanization, physical inactivity, and dietary transition) which promote obesity and insulin resistance but also environmental and genetic factors have not been well explored [2]. Data on the changes in the *β*-cell function and insulin resistance in the early stages of the disease process in African populations are scarce [21]. A few genetic studies performed in small groups from northern and western Africa had identified some polymorphism associated with diabetes but epigenetic factors which should play an important role in the disease onset are still unknown [22–24].

Other conditions like HIV and sickle cell diseases are also incriminated in the current epidemics of diabetes in Africa [25, 26].

Despite its epidemiological importance of describing diabetes face in Senegalese populations, this study has many limitations. Firstly, the cross-sectional design is not suitable for inferential analysis about causality or direction of association between diabetes and other cardiovascular risk factors. Secondly, incidence of diabetes could not be calculated to estimate the disease potential progression in the population.

## 5. Conclusion

This study shows that diabetes is frequent in northern region of Senegal. Urban settings are more concerned than rural areas and prevalence is higher among women. The awareness rate is very low among populations. Age, familial history of diabetes, and abdominal obesity are the main risk factors identified. Prevention program targeting both urban and rural populations are urgently needed in African countries in order to reduce the morbidity and mortality due to diabetes.

## Figures and Tables

**Table 1:** Clinical and biochemical characteristics of participants.

	All participants (*n* = 1027)		Urban areas (*n* = 572)		Rural areas (*n* = 455)		*p* value
	Crude	Adjusted^*∗*^	Crude	Adjusted^*∗*^	Crude	Adjusted^*∗*^	
Age (years)	48.0 ± 16.9 (18–87)		51.6 ± 15.7		43.5 ± 17.2		<0.01
Age group							<0.01
18–34 years	25.6%		16.0%		37.8%		
35–49 years	25.3%		26.1%		24.2%		
50–60 years	23.6%		28.0%		18.1%		
>60 years	25.5%		29.9%		19.9%		
School education	60.7%	52%	63.4%	47.5%	55.6%	45.8%	0.19
Family diabetes history	30.2%	28.3%	35.1%	29.6%	28.6%	26.4%	0.04
Fruits/vegetables consumption (≥3 portions/day)	39.0%	36.7%	42.3%	40.5%	34.6%	25.6%	0.05
Smoking	4.2%	2.9%	5.2%	3.1%	2.8%	1.7%	0.05
Physical inactivity	58.1%	53.5%	61.7%	56.2%	55.3%	48.6%	0.02
Hypertension	39.1%	32.1%	43.3%	36.8%	33.8%	29.2%	0.08
Body mass index (kg/m^2^)	26.3 ± 6.8	26.9 ± 1.6	27.9 ± 7.3	24.6 ± 1.3	24.1 ± 5.5	12.7 ± 5.1	0.03
Obesity	23.4%	20.5%	33.8%	31.6%	10.2%	9.4%	0.01
Waist circumference (cm)	90.6 ± 16.1	90.1 ± 8.2	94.4 ± 15.6	91.6 ± 10	86.0 ± 15.6	82.4 ± 8.5	0.01
Abdominal obesity	53.1%	48.6%	63.9%	59.5%	40.0%	33.4%	0.01
Total cholesterol (g/L)	2.18 ± 0.5	2.15 ± 0.1	2.25 ± 0.5	2.61 ± 3.2	2.10 ± 0.4	2.08 ± 1.1	<0.01
Hypercholesterolemia	56.0%	54.6%	62.5%	59.3%	46.7%	43.4%	0.53

^*∗*^Adjusted for age.

**Table 2:** Crude association between diabetes and risk factors (univariate analysis).

	Odds ratio	[95% confidence interval]	*p* value
Age group (<35 versus ≥35 yrs)	1.79	1.55–2.37	0.001
Female gender	1.14	1.05–3.28	0.045
Family history of diabetes	1.60	0.76–1.45	0.001
School education	0.88	0.70–1.00	0.038
Fruits/vegetables consumption (≥3/day)	0.95	0.65–2.57	0.244
Smoking	1.05	0.92–2.68	0.229
Physical inactivity	1.22	0.45–2.96	0.502
Hypertension	1.29	0.61–3.64	0.073
Obesity (BMI ≥ 30 kg/m^2^)	1.10	0.85–1.92	0.309
Abdominal obesity	1.66	1.40–2.03	0.001
Living in urban setting	1.40	1.10–2.47	0.039

BMI: body mass index.

**Table 3:** Adjusted association between diabetes and risk factors (multivariate regression analysis).

	Odds ratio	[95% confidence interval]	*p* value
Age group (<35 versus ≥35 yrs)	1.63	1.48–2.06	0.001
Female gender	1.42	0.75–1.84	0.108
Fruits/vegetables consumption (≥3/day)	0.87	0.25–1.43	0.326
Family history of diabetes	1.49	1.12–3.77	0.001
Physical inactivity	1.04	0.54–3.56	0.235
School education	1.02	0.33–1.92	0.164
Abdominal obesity	1.17	1.00–1.78	0.055
Living urban setting	1.27	0.65–2.34	0.073

*N* = 654, Pseudo-*R* ^2^ = 0.152.
